# Supplementary material for: The greenhouse gas impacts of converting food production in England and Wales to organic methods
Source: Nat Commun. 2019 Oct 22;10:4641. doi: 10.1038/s41467-019-12622-7 (PMC6805889; doi:10.1038/s41467-019-12622-7)
Supplement: Supplementary file 1 — Supplementary Information [file 41467_2019_12622_MOESM1_ESM.docx]

**Supplementary Information**

Supplementary Table 1 Production quantities for the baseline conventional and organic scenario, together with individual GHG emission factors for each commodity. Emissions from all crops needed to produce livestock commodities are included under each livestock commodity

|  | Crop production for direct human consumption | | | Emission factor  (CO_2e_ t^-1^) | |  | | Livestock commodities | | | Emission factor  (CO_2e_ t^-1^) | |
| --- | --- | --- | --- | --- | --- | --- | --- | --- | --- | --- | --- | --- |
|  | Conventional 2010 Baseline - '000 tonnes | Organic scenario - '000 tonnes | Conventional | | Organic | |  | | Conventional 2010 Baseline - '000 tonnes | Organic scenario - '000 tonnes | Conventional | Organic |
| Wheat | 6,935 | 3,572 | 0.42 | | 0.33 | | Sheep meat | | 184 | 235 | 20 | 19 |
| Barley | 1,034 | 605 | 0.38 | | 0.31 | | Beef | | 469 | 509 | 15 | 13 |
| Triticale & Rye | 0 | 14 | 0.52 | | 0.32 | | Pig meat | | 482 | 135 | 4 | 4 |
| Oats | 161 | 403 | 0.35 | | 0.37 | | Eggs | | 504 | 152 | 3 | 3 |
| Potatoes | 3,275 | 4,912 | 0.13 | | 0.14 | | Poultry meat | | 716 | 178 | 5 | 5 |
| OSR | 518 | 46 | 0.89 | | 0.76 | | Milk | | 10,302 | 6,511 | 1 | 1 |
| Sugar beet | 4,020 | 3,629 | 0.06 | | 0.06 | |  | |  |  |  |  |
| Cabbage | 248 | 170 | 0.07 | | 0.05 | |  | |  |  |  |  |
| Carrots | 619 | 928 | 0.04 | | 0.04 | |  | |  |  |  |  |
| Onions | 345 | 518 | 0.15 | | 0.16 | |  | |  |  |  |  |
| Source C:\Z\Nature Paper\Revisions\V2\Uncertainty\Data_sets AGW.xlsx GHG AGW | | | | | | | | | | | | |

Supplementary Table 2 Human-edible food output in England and Wales under 100% organic and conventional scenarios expressed as total Metabolisable Energy (ME) and Crude Protein (CP) production by Eatwell^1^ food group

## Supplementary Table 3 Manure production estimates based on standard values for the UK and scaled by yield (dairy cows only). C in manure is calculated on the basis of Total Solid (TS) and Volatile Solid (VS) content data[^3^](#_ENREF_3)

Supplementary Table 4 Data sources used to represent organic production systems in the OLUM and Agri-LCA models


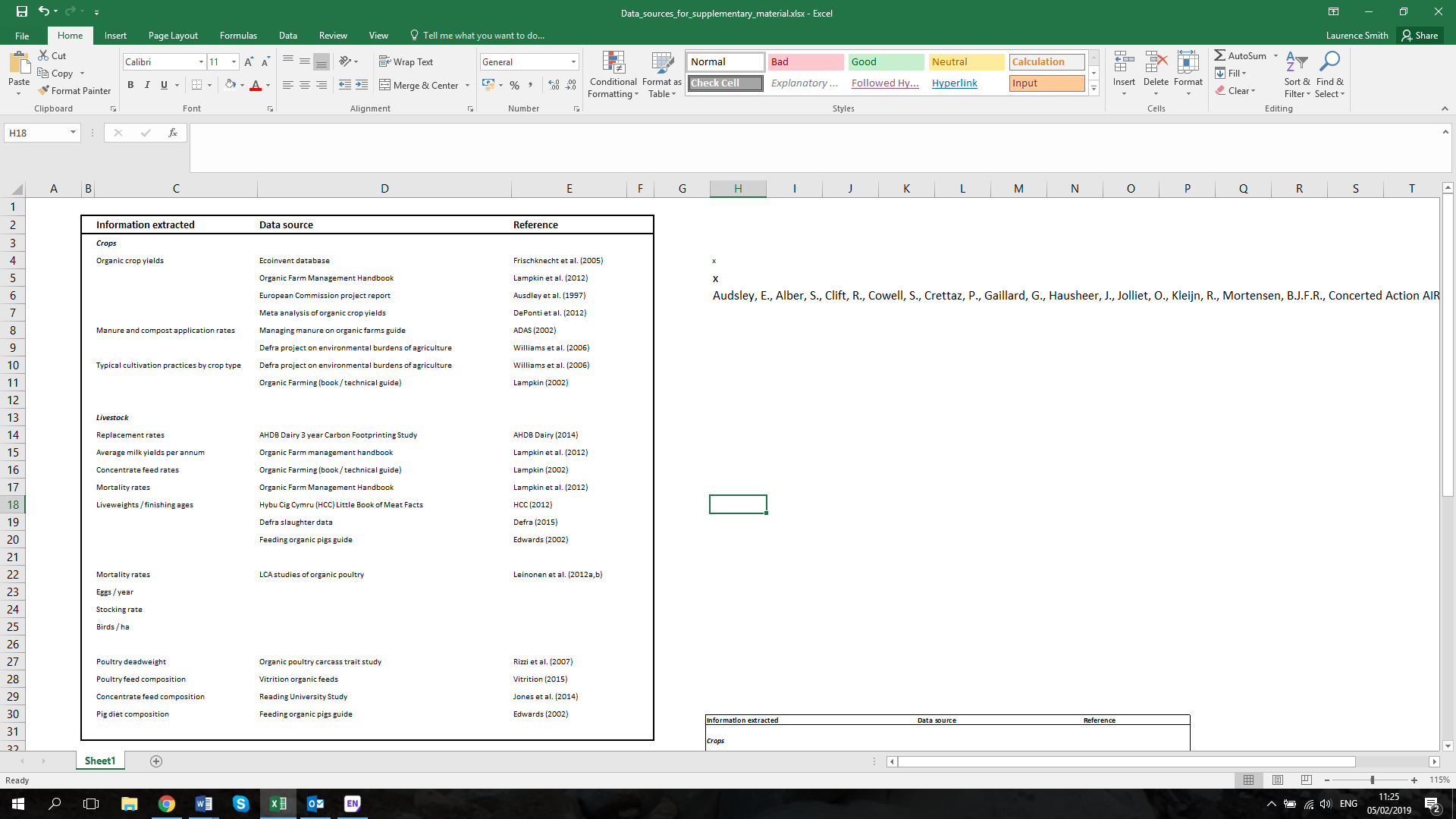


Note to table: Organic farm structure data (i.e. land areas by crop type and stocking rates) were drawn from a panel of organic farms in the Farm Business Survey for England and Wales (i.e. values for the years 2009/10 – 2011/12 by Robust Farm Type, as reported by Moakes *et al.* (2012, 2014).

Supplementary Table 5 Non-organic production and import / export data sources used in this study


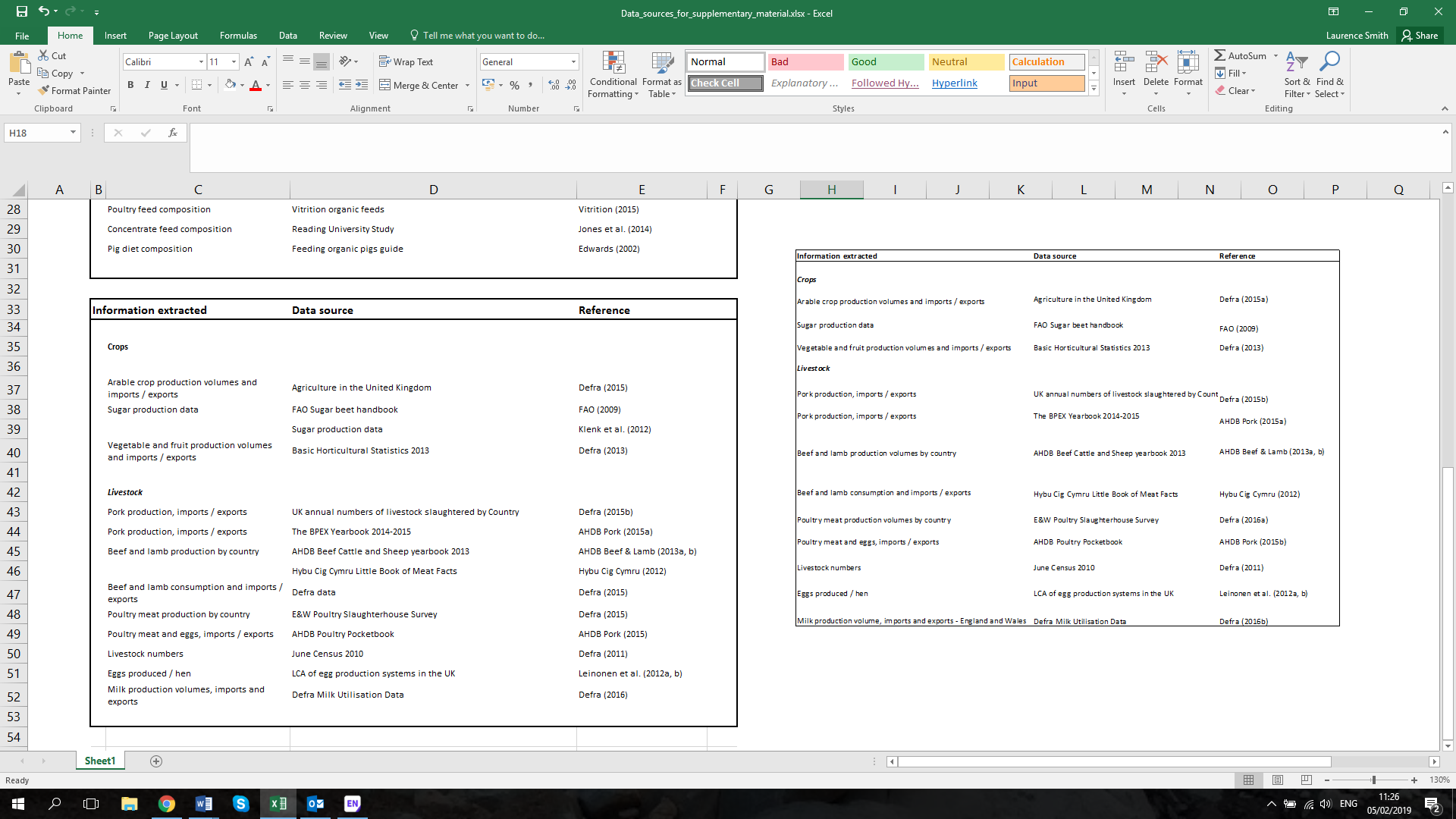


Supplementary Table 6 Land use per tonne values applied in this study to calculate overseas land requirements for imports. Note that the land use requirements refer only to the *non-forage* component of the diet. Yield data reported in de Ponti et al.[^32^](#_ENREF_32) are lower than estimates in Williams et al.[^3^](#_ENREF_3) which leads to the greater overseas land-use area requirements for poultry, eggs, beef and sheep from ‘other literature’.

|  | Overseas land use requirements (ha t^-1^) | | |
| --- | --- | --- | --- |
|  | Non-organic from Williams et al.^3^ | Organic from Williams et al.^3^ | Organic from other literature ^23,32,46,50^ |
| Pork (dressed carcass) | 0.7 | 1.3 | 1.0 |
| Poultry (dressed carcass) | 0.6 | 1.4 | 2.5 |
| Eggs | 0.7 | 1.5 | 1.7 |
| Milk ('000 litres fat adjusted) | 0.04 | 0.07 | 0.07 |
| Beef (dressed carcass) | 0.3 | 0.5 | 1.2 |
| Sheep (dressed carcass) | 0.2 | 0.4 | 1.0 |

Supplementary Table 7 Overall uncertainties (expressed as coefficients of variation) from the GHG emissions of the sum of crops and livestock resulting from domestic and overseas production, transport for imports, emissions from LUC overseas (positive) and C sequestration (negative)

|  | **Conventional** | **Organic** |
| --- | --- | --- |
| Crops, domestic | 10.6% | 10.4% |
| Crops, overseas | 12.2% | 11.9% |
|  |  |  |
| Livestock, domestic | 10.7% | 10.7% |
| Livestock, overseas | 12.3% | 12.3% |
|  |  |  |
| Transport | 10.0% | 10% |
|  |  |  |
| LUC Loss |  | 17.3% |
| LUC Sequestration Lower |  | 23.8% |
| LUC Sequestration Upper |  | 86% |

## Supplementary references

1. ADAS. *Managing Manure on Organic Farms* (ADAS, Gleadthorpe, UK, 2002).
2. AHDB Beef and Lamb. *UK Yearbook 2013 – Cattle* (AHDB, Stoneleigh Park, UK, 2013).
3. AHDB Beef and Lamb. *UK Yearbook 2013 – Sheep* (AHDB, Stoneleigh Park, UK, 2013).
4. AHDB Dairy. *Greenhouse gas emissions on British dairy farms* (AHDB, Stoneleigh Park, UK, 2014).
5. AHDB Pork. *Poultry Pocketbook* (AHDB, Stoneleigh Park, UK, 2015).
6. AHDB Pork. *The BPEX Yearbook 2014-2015* (AHDB, Stoneleigh Park, UK, 2015).
7. Audsley, E., et al. *Concerted Action AIR3-CT94-. European Commission, DG VI Agriculture, Harmonisation of environmental life cycle assessment for agriculture* (DG Agriculture Brussels, 1997)
8. Defra. *Agriculture in the United Kingdom* (Defra, London, 2015).
9. Defra. *Basic Horticultural Statistics* (Defra, London, 2013).
10. Defra. *June Survey of Agriculture and Horticulture* (Defra, London, 2011).
11. Defra. *Monthly Utilisation of Milk by Dairies in England and Wales* (Defra, London, 2016).
12. Defra. *Slaughterhouse Surveys Data* (Defra, London, 2015).
13. Edwards, S. *Feeding Organic Pigs: a handbook* (Newcastle University, UK, 2002).
14. FAO. *Sugar Beet Agribusiness Handbook* (FAO, Rome, 2009).
15. Frischknecht, R. et al. The Ecoinvent database: Overview and methodological framework (7 pp). Int. J. Life. Cycle. Assess. 10, 3-9 (2005).
16. Hybu Cig Cymru. *Little Book of Meat Facts* (Hybu Cig Cymru, Aberystwyth, Wales, 2012).
17. Jones, P., Thomas, D., Hazzledine, M. & Rymer, C. *Replacing soya in livestock feeds with UK-grown protein crops: prospects and implications* (University of Reading, UK, 2014).
18. Klenk, I., Lanquist, B. & de Imana, O., 2012. The Product Carbon Footprint of EU Beet Sugar. Sugar. Ind. 137.
19. Lampkin, N. *Organic Farming* (Old Pond Publishing Ltd., Ipswich, UK, 2002).
20. Lampkin, N., Measures, M. & Padel, S. *2011/12 Organic Farm Management Handbook* (The Organic Research Centre, Newbury, UK, 2012).
21. Leinonen, I., Williams, A. G., Wiseman, J., Guy, J. & Kyriazakis, I. Predicting the environmental impacts of chicken systems in the United Kingdom through a life cycle assessment: Broiler production systems. Poult. Sci. 91, 8-25 (2012).
22. Leinonen, I., Williams, A. G., Wiseman, J., Guy, J. & Kyriazakis, I. Predicting the environmental impacts of chicken systems in the United Kingdom through a life cycle assessment: Egg production systems. Poult. Sci. 91, 26-40 (2012).
23. Moakes, S., Lampkin, N. & Gerrard, C. L. *Organic farm incomes in England and Wales 2012/13* (University of Wales, Aberystwyth, UK, 2014).
24. Moakes, S., Lampkin, N. & Gerrard, C. L. *Organic farm incomes in England and Wales 2010/11* (University of Wales, Aberystwyth, UK, 2012).
25. Rizzi, C., Marangon, A. & Chiericato, G. Effect of genotype on slaughtering performance and meat physical and sensory characteristics of organic laying hens. Poul. Sci. 86, 128-135 (2007).
26. Vitrition Organic Feeds. *Feed composition data* (Vitrition, Boroughbridge, UK, 2015).
27. Williams, A. G., Audsley, E., Sandars, D. L. *Determining the environmental burdens and resource use in the production of agricultural and horticultural commodities* (Defra, London, 2006).
