# Supplementary material for: The greenhouse gas impacts of converting food production in England and Wales to organic methods
Source: Nat Commun. 2019 Oct 22;10:4641. doi: 10.1038/s41467-019-12622-7 (PMC6805889; doi:10.1038/s41467-019-12622-7)
Supplement: Supplementary file 3 — Reporting Summary [file 41467_2019_12622_MOESM3_ESM.pdf]

nature research

Corresponding author(s): Prof. Guy Kirk

# Reporting Summary

Nature Research wishes to improve the reproducibility of the work that we publish. This form provides structure for consistency and transparency in reporting. For further information on Nature Research policies, see Authors & Referees and the Editorial Policy Checklist.

## Statistical parameters

When statistical analyses are reported, confirm that the following items are present in the relevant location (e.g. figure legend, table legend, main text, or Methods section).

| n/a | Confirmed | |
|---|---|---|
| ☒ | ☐ | The exact sample size ($n$) for each experimental group/condition, given as a discrete number and unit of measurement |
| ☒ | ☐ | An indication of whether measurements were taken from distinct samples or whether the same sample was measured repeatedly |
| ☒ | ☐ | The statistical test(s) used AND whether they are one- or two-sided<br>*Only common tests should be described solely by name; describe more complex techniques in the Methods section.* |
| ☒ | ☐ | A description of all covariates tested |
| ☒ | ☐ | A description of any assumptions or corrections, such as tests of normality and adjustment for multiple comparisons |
| ☒ | ☐ | A full description of the statistics including central tendency (e.g. means) or other basic estimates (e.g. regression coefficient) AND variation (e.g. standard deviation) or associated estimates of uncertainty (e.g. confidence intervals) |
| ☒ | ☐ | For null hypothesis testing, the test statistic (e.g. $F$, $t$, $r$) with confidence intervals, effect sizes, degrees of freedom and $P$ value noted<br>*Give P values as exact values whenever suitable.* |
| ☒ | ☐ | For Bayesian analysis, information on the choice of priors and Markov chain Monte Carlo settings |
| ☒ | ☐ | For hierarchical and complex designs, identification of the appropriate level for tests and full reporting of outcomes |
| ☒ | ☐ | Estimates of effect sizes (e.g. Cohen's $d$, Pearson's $r$), indicating how they were calculated |
| ☐ | ☒ | Clearly defined error bars<br>*State explicitly what error bars represent (e.g. SD, SE, CI)* |

*Our web collection on statistics for biologists may be useful.*

## Software and code

Policy information about availability of computer code

| | |
|---|---|
| Data collection | The programming language GAMS (General Algebraic Modelling System, www.gams.com) was used for the construction of the Optimal Land Use Model (OLUM). The model code is available at: https://www.dropbox.com/sh/hgfu2rby0ntqgm2/AABX5i1yaLHw76_K0B9T6l-Ma?dl=0<br>The Agri-LCA models are available at:<br>https://www.dropbox.com/s/9mhfrr9heiujn2q/AGRILCA_model_2013.zip?dl=0 |
| Data analysis | A graphical interface was written in Microsoft Visual Basic (VB) to allow rapid and easy interrogation and analysis of the of the Agri-LCA models |

For manuscripts utilizing custom algorithms or software that are central to the research but not yet described in published literature, software must be made available to editors/reviewers upon request. We strongly encourage code deposition in a community repository (e.g. GitHub). See the Nature Research guidelines for submitting code & software for further information.

## Data

Policy information about availability of data

All manuscripts must include a data availability statement. This statement should provide the following information, where applicable:

- Accession codes, unique identifiers, or web links for publicly available datasets
- A list of figures that have associated raw data
- A description of any restrictions on data availability

The underlying data can be accessed at: https://figshare.com/s/91b7d86aa11b8ac51cf2
Additional data and model code will be made available by the authors on request.

# Field-specific reporting

Please select the best fit for your research. If you are not sure, read the appropriate sections before making your selection.

☐ Life sciences ☐ Behavioural & social sciences ☒ Ecological, evolutionary & environmental sciences

For a reference copy of the document with all sections, see nature.com/authors/policies/ReportingSummary-flat.pdf

# Ecological, evolutionary & environmental sciences study design

All studies must disclose on these points even when the disclosure is negative.

**Study description**

We apply a large-scale linear programming model and a separate Life Cycle Assessment based model to assess the production and environmental impacts associated with a 100% conversion to organic farming in England and Wales.

**Research sample**

Optimal Land Use Model (OLUM) data sources:

Land availability by farm type and land class were determined using official national survey data (i.e. the Farm Business Survey and June Agricultural Survey). Crop yields were derived through application of the NDICEA model (Nitrogen Dynamics In Crop Rotations in Ecological Agriculture). N fixation data and crop N contents per tonne were derived from technical guides, Defra Fertiliser Recommendations and the nutrient budgeting software PLANET (version 2.2). Atmospheric nitrogen deposition values were extracted from national pollution data downloaded from the Centre for Ecology and Hydrology (CEH) website (http://www.pollutantdeposition.ceh.ac.uk/). Volumes of manure-N supplied by each livestock type were derived from reference data for organic farming inspections, plus data derived from guidance on managing manure on organic farms, and information on Nitrogen Vulnerable Zones (NVZ) management in the UK.

Agri-LCA data sources:

Crop yields and (organic and non-organic) practice data were derived from an inventory of materials and processes from technical information sources which were combined with expert knowledge, inputs from an EU harmonisation study and data from the Ecoinvent database provided within the SimaPro platform. The impacts of producing crops and livestock products in the 2010 non-organic baseline were derived through application of animal production data from standard sources which define the structure of the current industry (e.g. % upland vs lowland sheep) and technical performance by livestock type with respect to factors such as daily liveweight gain and feed conversion ratios. For assessing the relative impact of the organic scenario, farm structures were derived from the OLUM (e.g. with respect to proportion of livestock on upland/lowland areas) and technical performance derived from a range of data sources. Annual topsoil carbon sequestration estimates for each hectare of organically-managed land were applied using estimates reported within a recent meta analysis.

Total amounts of imported crop and livestock product were accessed from Defra. Estimates of total land-use overseas were derived through average yield data from Eurostat, a recent meta-analysis on organic crop yields and from results for an LCA of milling wheat grown in Canada. Land-use requirements per tonne of livestock-product were derived from the Agri-LCA and from data within recent studies on the environmental burdens of imported lamb produced in New Zealand. Land Use Change values were calculated using values from the PAS2050 Specification. Organic oilseed rape yields were extracted from published survey data and organic sunflower yields from a long-term experiment in central Italy.

**Sampling strategy**

Representative official national surveys reporting on the economic performance of organic farming were used to develop organic farm structures for use in the models applied in this study (i.e. the organic sub-sample of the Defra Farm Business Survey, FBS). Where crop rotations were drawn from literature sources, advice was sought from advisors registered with the Institute for Organic Training and Advice on the representativeness of the cropping sequences and comparisons made with actual crop areas reported in the FBS sample over three years (2010-2012). Although there are some differences by crop type between 'book values' and survey data, the differences are generally in the region of 15–20% of the total farmed area. In view of the wide variation between the rotations on individual farms, this is an acceptable margin of error and the rotations applied here can be considered to be broadly representative of organic farms. For some farm-types data gathered in previous research were used, e.g. for specialist pigs and poultry, as these systems are under-represented or missing from the organic FBS sample

**Data collection**

Data were collected by the lead author and recorded in databases (Microsoft Access) for analysis in Microsoft Excel and GAMS

| | |
|---|---|
| Timing and spatial scale | Data were collected by the lead author over the years 2012-2016. Soil, rainfall and farm-type coverage data were collected on a 5km grid square basis |
| Data exclusions | A land cover map was used to remove extraneous areas from the land-use dataset (e.g. urban conurbations, bracken, sea estuary, woodland) |
| Reproducibility | Methods code and data are provided with the manuscript and should be easily reproducible |
| Randomization | Farms selected for inclusion within the FBS are selected at random with uniform probability. Addresses are obtained from the June Survey of Agriculture. The refusal rate is high; around 90% of those approached who are in scope. The potential population of non-respondents may have significantly different characteristics from the potential population of respondents, leading to bias in the estimates of the full population. Calibration weighting is used to reduce this bias, but is unlikely to completely remove it. |
| Blinding | Blinding was not relevant to this study as we used existing survey data within research archives and/or published literature to calibrate the models |

Did the study involve field work? ☐ Yes ☒ No

# Reporting for specific materials, systems and methods

## Materials & experimental systems

| n/a | Involved in the study |
|---|---|
| ☒ | ☐ Unique biological materials |
| ☒ | ☐ Antibodies |
| ☒ | ☐ Eukaryotic cell lines |
| ☒ | ☐ Palaeontology |
| ☒ | ☐ Animals and other organisms |
| ☒ | ☐ Human research participants |

## Methods

| n/a | Involved in the study |
|---|---|
| ☒ | ☐ ChIP-seq |
| ☒ | ☐ Flow cytometry |
| ☒ | ☐ MRI-based neuroimaging |
